# Association Between Pulmonary Hypertension and Its Effect on 30-Day Mortality, Readmission, and Cost After Transcatheter Aortic Valve Replacement: A Multicenter Study

**DOI:** 10.7759/cureus.40976

**Published:** 2023-06-26

**Authors:** Mansoor Ahmad, Juan Del Cid Fratti, Mena Henien, Kailash Pant, Matthew P Wattelet, Daniel Whorf, Brett C Austin, Minchul Kim, Marco Barzallo, Sudhir Mungee

**Affiliations:** 1 Cardiology, University of Illinois College at Chicago Peoria, Peoria, USA

**Keywords:** cost, icu days, 30-day mortality, pulmonary hypertension, transcatheter aortic valve replacement

## Abstract

Background

Pulmonary hypertension (PH) is commonly seen in patients with severe aortic stenosis. Transcatheter aortic valve replacement (TAVR) has been shown to improve PH, however, its impact on clinical outcomes and cost remains unclear.

Methods

We did a multicenter, retrospective analysis of patients undergoing TAVR in our system between December 2012 to November 2020. The initial sample size was 1356. We excluded patients with prior history of heart failure, with a left ventricular ejection fraction of 40% or less, and patients who had active symptoms of heart failure within two weeks of the procedure. Patients were divided into four groups based on their pulmonary pressures, using right ventricular systolic pressure (RVSP) as a surrogate for PH. Groups included patients with normal pulmonary pressures (<35mmHg), mild (35-45mmHg) moderate (46-60mmHg), and severe PH (>60mmHg). Primary outcomes included 30-day mortality and readmission. Secondary outcomes included length of ICU stay and cost of admission. We used Chi-square and T-tests for demographic analysis of categorical continuous variables respectively. Adjusted regression was used for the reliability of correlation between variables. Multivariate analysis was used for final outcomes.

Results

Final sample size was 474. Average age was 78.9 years (SD: 8.2, 53% Male). Thirty-one percent (n=150) had normal pulmonary pressures, 33% (n=156) had mild PH, 25% (n=122) had moderate and 10% (n=46) had severe PH. Patients with history of hypertension (p-value<0.001), diabetes (p-value<0.001), chronic lung disease (p-value=0.006) and those on supplemental oxygen (p-value=0.046), had significantly higher proportion of patients with moderate and severe PH.

We found significantly higher odds of 30-day mortality in patients with severe PH (OR: 6.77, CI: 1.09-41.98: p-value 0.04) compared with normal or mild PH. There was no significant difference in 30-day readmission (p-value=0.859) between the four groups. Cost did not change with severity of PH (Avg: $261,075: p-value=0.810). Patients with severe PH spent a significantly higher number of hours in ICU, compared with the other three groups (Mean: 18.2, p=value <0.001).

Conclusions

Severe pulmonary hypertension significantly increased the odds of 30-day mortality and ICU stay in TAVR patients. We did not see any significant difference in 30-day readmission and cost of admission, based on PH severity.

## Introduction

It is estimated that approximately 1% of the global population is affected by pulmonary hypertension (PH) [1]. PH is commonly associated with aortic stenosis, and PH in patients with severe aortic stenosis is correlated with increased mortality rates [2]. Prior studies have also suggested greater costs and readmission rates for patients with PH [3,4]. Thus, it is important to determine how medical interventions can improve outcomes for patients with PH while simultaneously reducing costs.

Patients with preexisting pulmonary hypertension are considered high risk for undergoing aortic valve replacement. Patients with aortic stenosis who undergo transcatheter aortic valve replacement (TAVR) demonstrate reduced PH levels [5]. However, the effects of preexisting PH on clinical outcomes and cost in patients undergoing TAVR remain unclear.

Prior studies have examined clinical outcomes among patients with PH who undergo TAVR. These studies stratified patients according to the severity of PH and demonstrated higher mortality rates with greater pulmonary arterial pressures [5,6]. However, neither of these studies examined how the severity of PH affects morbidity, short-term mortality and total cost of admission.

This study seeks to clarify how PH severity affects 30-day mortality rates, 30-day readmission rates, the total time in the intensive care unit (ICU), and cost of admission in patients undergoing TAVR.

## Materials and methods

A retrospective study of 1356 patients who underwent TAVR at Saint Francis Medical Center between December 2012 and November 2020 was conducted. IRB approval was granted by the University of Illinois at Chicago IRB Department.

The initial sample size was 1356. Patients with a prior history of heart failure, left ventricular ejection of 40% or less, or active heart failure symptoms within two weeks of the procedure were excluded from the study. After excluding these patients, a final sample size of 474 was obtained.

The clinical variables studied included age, gender, race, smoking habits, history of hypertension, history of diabetes, current use of dialysis, history of chronic lung disease, current supplemental oxygen use, current atrial fibrillation or flutter, history of peripheral artery disease, history of prior aortic valve replacement, current use of a pacemaker, and history of coronary artery bypass graft surgery (CABG).

Patients were divided into four groups based on pulmonary pressures, estimated on the Echocardiogram prior to the procedure. The groups included: normal pulmonary pressures (<35mmHg), mild PH (35-45mmHg), moderate PH (46-60mmHg), and severe PH (>60mmHg).

Outcome comparison

Thirty-day mortality and readmission were primary variables, while length of ICU admission and cost were secondary variables.

Statistical analysis

Clinical data and baseline characteristics across the four groups were compared. Percentages and proportions were used to represent categorical variables, while mean ± standard deviation (SD) was used to represent continuous variables. Chi-square tests were used to analyze categorical variables, and t-tests were used to analyze continuous variables. Adjusted regression was applied to ensure the reliability of correlations between clinical variables. Multivariate analysis was performed to determine 30-day mortality, 30-day readmission, mean number of ICU hours, and cost. All calculations were performed using Stata software (StataCorp., College Station, TX, USA).

## Results

Sample demographics

Out of 474 patients, 150 had normal pulmonary pressure, 156 had mild pulmonary hypertension, 122 had moderate pulmonary hypertension, and 46 had severe pulmonary hypertension. The average age of the entire sample was 78.9 years (SD: 8.2). Patients in the severe pulmonary hypertension group had a significantly higher proportion of patients with primary hypertension (97.8 vs. 83.3%: p<0.001), with diabetes (56.5 vs 26.7%: p<0.001), on current dialysis (10.9 vs 1.3%: p<0.001), with chronic lung disease (50 vs 26%: p=0.006), on supplemental oxygen (HmO2; 10.9 vs 5.3%: p=0.046), with an atrial fibrillation or flutter (58.7 vs 23.3%: p<0.001), and with a prior history of CABG surgery (10.9 vs 9.3%: p=0.006) had a higher percentage of patients with moderate and severe pulmonary hypertension (Table 1).

**Table 1:** Baseline Demographics * A t-test was used for descriptive variables and a Chi-square test was used for categorical variables. HmO2: Supplemental oxygen, AFibFlutter: Atrial fibrillation or atrial flutter, PAD: Peripheral artery disease, PCI: Percutaneous coronary intervention, CABG: Coronary artery bypass graft

Variables	All (n=474)		Normal (n=150)		Mild (n=156)		Moderate (n=122)		Severe (n=46)		P value*
	Frequency	%	Frequency	%	Frequency	%	Frequency	%	Frequency	%	
Age											0.653
<70	56	11.8	12	8.0	20	12.8	17	13.9	7	15.2	
70 - 85	314	66.2	104	69.3	99	63.5	82	67.2	29	63.0	
>85	104	21.9	34	22.7	37	23.7	23	18.9	10	21.7	
Gender											0.366
Male	252	53.2	87	58.0	84	53.9	58	47.5	23	50.0	
Female	222	46.8	63	42.0	72	46.2	64	52.5	23	50.0	
Race											0.180
White	468	98.7	150	100.0	154	98.7	119	97.5	45	97.8	
Black	3	0.6	0	0.0	0	0.0	2	1.6	1	2.2	
Hispanic	2	0.4	0	0.0	2	1.3	0	0.0	0	0.0	
Other	1	0.2	0	0.0	0	0.0	1	0.8	0	0.0	
Smoker											0.318
No	442	93.3	141	94.0	149	95.5	110	90.2	42	91.3	
Yes	32	6.8	9	6.0	7	4.5	12	9.8	4	8.7	
Hypertension											0.000
No	42	8.9	25	16.7	11	7.1	5	4.1	1	2.2	
Yes	432	91.1	125	83.3	145	93.0	117	95.9	45	97.8	
Diabetes											0.000
No	279	58.9	110	73.3	96	61.5	53	43.4	20	43.5	
Yes	195	41.1	40	26.7	60	38.5	69	56.6	26	56.5	
Current dialysis											0.001
No	463	97.7	148	98.7	153	98.1	121	99.2	41	89.1	
Yes	11	2.3	2	1.3	3	1.9	1	0.8	5	10.9	
Chronic lung disease											0.006
None	312	65.8	111	74.0	110	70.5	68	55.7	23	50.0	
Mild	82	17.3	22	14.7	25	16.0	26	21.3	9	19.6	
Moderate	41	8.7	8	5.3	14	9.0	14	11.5	5	10.9	
Severe	39	8.2	9	6.0	7	4.5	14	11.5	9	19.6	
HmO2											0.047
No	439	92.6	142	94.7	149	95.5	107	87.7	41	89.1	
Yes	35	7.4	8	5.3	7	4.5	15	12.3	5	10.9	
AFibFlutter											0.000
No	323	68.1	115	76.7	114	73.1	75	61.5	19	41.3	
Yes	151	31.9	35	23.3	42	26.9	47	38.5	27	58.7	
Prior PAD											0.070
No	301	63.5	98	65.3	103	66.0	79	64.8	21	45.7	
Yes	173	36.5	52	34.7	53	34.0	43	35.3	25	54.4	
Prior Aortic Valve											0.844
No	450	94.9	142	94.7	150	96.2	115	94.3	43	93.5	
Yes	24	5.1	8	5.3	6	3.9	7	5.7	3	6.5	
Prior PCI											0.724
No	338	71.3	112	74.7	108	69.2	85	69.7	33	71.7	
Yes	136	28.7	38	25.3	48	30.8	37	30.3	13	28.3	
Pacemaker											0.332
No	447	94.3	144	96.0	149	95.5	112	91.8	42	91.3	
Yes	27	5.7	6	4.0	7	4.5	10	8.2	4	8.7	
Prior Cabg											0.006
No	418	88.2	136	90.7	144	92.3	97	79.5	41	89.1	
Yes	56	11.8	14	9.3	12	7.7	25	20.5	5	10.9	

Pulmonary hypertension vs 30-day mortality, 30-day readmission, charge, and ICU hours

Patients with severe pulmonary hypertension exhibited higher 30-day mortality (OR: 6.77, CI: 1.09-41.98: p-value=0.04) when compared to patients with normal or mild pulmonary pressure. Patients with severe pulmonary hypertension also spent longer hours in the ICU than patients with normal, mildly elevated, or moderately elevated pulmonary pressure (mean: 18.2, p-value<0.001). However, there was no significant difference in 30-day readmission rates (Avg: 10.0%, p-value=0.859) and cost (Avg: $261,075: p-value=0.810) between the four groups.

According to Table 2, patients with severe PH had higher 30-day mortality rates than the other groups (p=0.028), but no significant difference was found in 30-day readmission rates (p=0.859), total charge (p=0.810), and total ICU hours (p=0.941).

**Table 2:** Effect of Pulmonary Hypertension on 30-Day Mortality, 30-Day Readmission, Charge, and ICU Hours ICU: Intensive care unit

Variables	All (n=474)		Normal (n=150)		Mild (n=156)		Moderate (n=122)		Severe (n=46)		P value*
	Frequency	%	Frequency	%	Frequency	%	Frequency	%	Frequency	%	
30-day mortality											0.028
No	466	98.3	150	100.0	153	98.1	120	98.4	43	93.5	
Yes	8	1.7	0	0.0	3	1.9	2	1.6	3	6.5	
30-day readmission											0.859
No	429	90.5	135	90.0	142	91.0	109	89.3	43	93.5	
Yes	45	9.5	15	10.0	14	9.0	13	10.7	3	6.5	
	Mean	SD	Mean	SD	Mean	SD	Mean	SD	Mean	SD	
Charge	257788	51916	261075	68430	255592	46239	256422	39003	258138	36229	0.810
ICU hours	16.0	25.1	15.5	30.9	15.9	19.7	15.9	19.6	18.2	32.8	0.941

**Figure 1:**
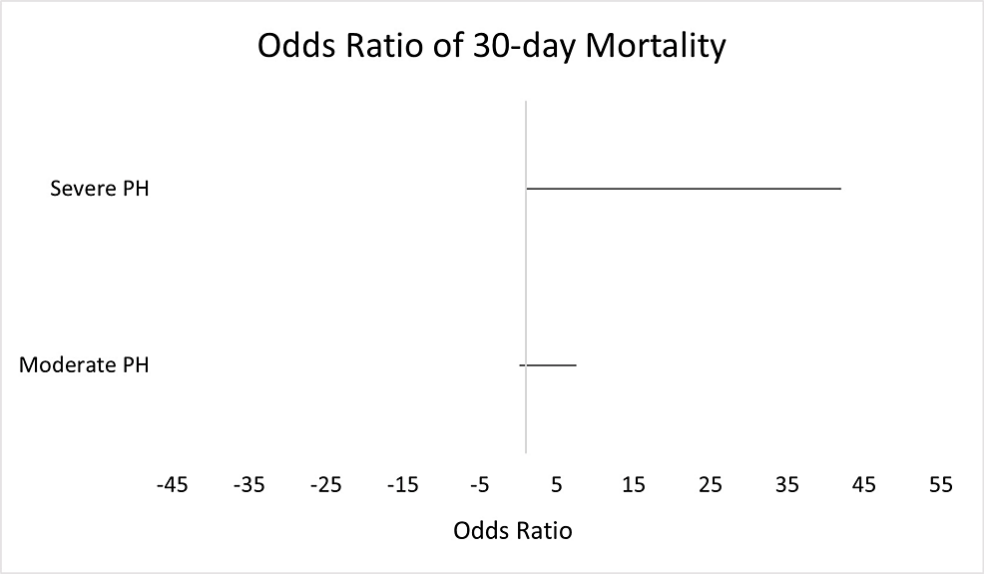
Odds Ratio of 30-Day Mortality in Patients With Severe and Moderate Pulmonary Hypertension (PH)

As seen in Figure 1, patients with severe pulmonary hypertension had a higher 30-day mortality rate when compared to patients with normal and mild pulmonary pressure (OR: 6.77, CI: 1.09-41.98: p-value=0.04). Patients with moderate pulmonary pressure did not demonstrate statistically significant increases in 30-day mortality (OR: 1.07, CI: 0.15-7.53, p-value=0.943).

**Figure 2:**
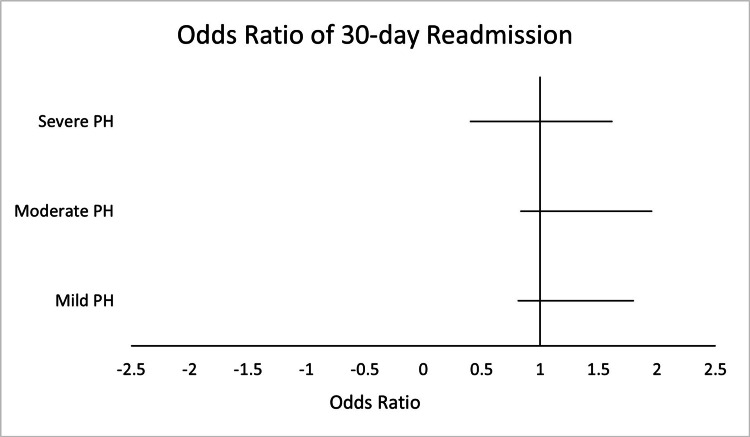
Odds Ratio for Multivariate Regression of 30-Day Readmission in Patients With Severe, Moderate, and Mild Pulmonary Hypertension (PH)

We did not find any significant differences in 30-day readmission between the four groups (Figure 2). Patients with severe PH had lower odds of being readmitted to hospital in 30 days compared with patients with normal pulmonary pressures (OR: 0.4, CI: 0.1-1.62, p-value=0.202).

**Figure 3:**
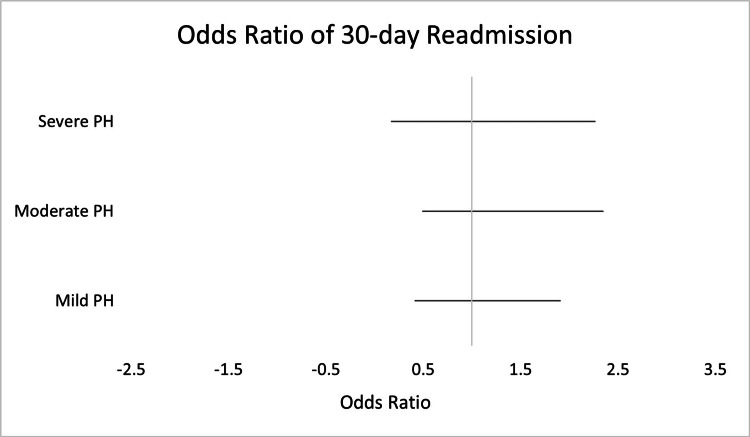
Odds Ratio for Univariate Regression of 30-Day Readmission in Patients With Severe, Moderate, and Mild Pulmonary Hypertension (PH)

Univariate regression model also did not find any significant differences in 30-day readmission between the four groups (Figure 3). Patients with severe PH had lower odds of being readmitted to hospital in 30 days compared with patients with normal pulmonary pressures (OR: 0.63, CI: 0.17-2.27, p-value=0.478).

**Figure 4:**
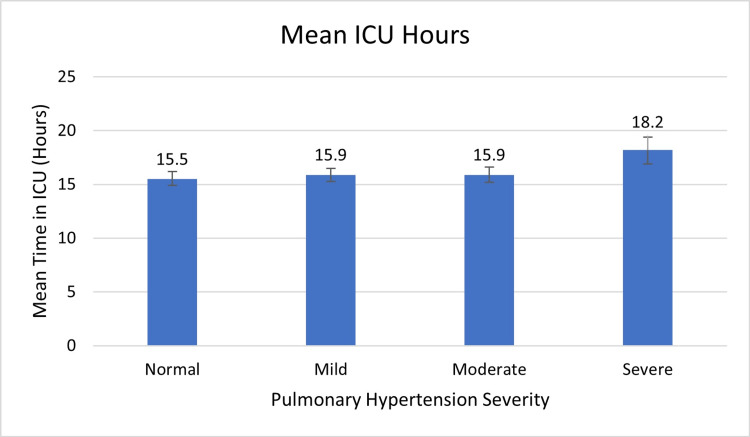
Pulmonary Hypertension Severity vs ICU Hours ICU: Intensive care unit

There was no significant difference in the time spent in the ICU amongst the four groups (Figure 4). On average, patients with severe PH spent slightly more hours in the ICU than the other groups (mean: 16.2 vs 15.5 hours: p-value=0.941).

According to Table 3, patients with severe pulmonary hypertension spent more time in the ICU than patients with normal, mildly elevated, or moderately elevated pulmonary pressure (mean: 18.2 vs 15.5, p-value<0.001).

**Table 3:** Univariate Regression Comparing Mean Number of Hours Spent in the ICU Among Patients With Normal, Mildly Elevated, Moderately Elevated, and Severely Elevated Pulmonary Pressure PH: Pulmonary hypertension

PH Groups	Predicted mean	95% CI		Difference	95% CI		P value
PH (Ref: Normal)	15.5	14.9	16.2				
Mild	15.9	15.3	16.5	0.4	-0.5	1.3	0.402
Moderate	15.9	15.2	16.6	0.4	-0.6	1.3	0.431
Severe	18.2	16.9	19.4	2.6	1.3	4.0	0.000

As described in Table 4, patients with moderate pulmonary hypertension spent less time in the ICU than patients with normal, mildly elevated, or severely elevated pulmonary pressure (mean: 14.8 vs 16.8, p-value<0.001).

**Table 4:** Multivariate Regression Comparing Mean Number of Hours Spent in the ICU Among Patients With Normal, Mildly Elevated, Moderately Elevated, and Severely Elevated Pulmonary Pressure PH: Pulmonary hypertension

PH Groups	Coefficient	95% CI		P value	Predicted mean	95% CI		Difference	95% CI		P value
PH (Ref: Normal)					16.8	16.1	17.5				
Mild	-0.02	-0.08	0.04	0.445	16.4	15.7	17.1	-0.4	-1.3	0.6	0.445
Moderate	-0.13	-0.19	-0.06	0.000	14.8	14.1	15.5	-2.0	-3.0	-1.0	0.000
Severe	-0.04	-0.13	0.04	0.320	16.1	14.9	17.2	-0.7	-2.1	0.7	0.315

**Figure 5:**
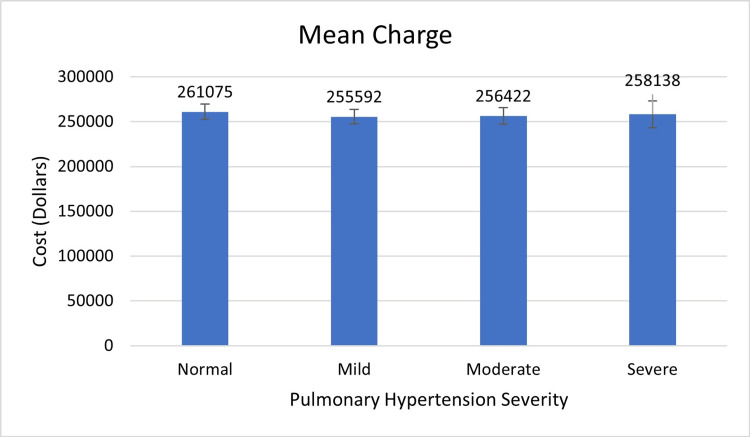
Pulmonary Hypertension Severity vs Cost

According to Figure 5, there was no significant difference in cost amongst the four groups (p-value=0.810). On average, patients with severe PH had slightly lower costs than the other groups (mean: $258,138 vs 261,075: p-value=0.738).

There was no difference in the mean cost between patients with normal, mildly elevated, moderately elevated, or severely elevated pulmonary pressure (p=0.810) (Table 5). On average, patients with severe PH had slightly lower costs than the other groups (mean: $258,138 vs 261,075: p-value=0.738).

**Table 5:** Univariate Regression Comparing Mean Cost Among Patients With Normal, Mildly Elevated, Moderately Elevated, and Severely Elevated Pulmonary Pressure PH: Pulmonary hypertension

PH Groups	Predicted mean	95% CI		Difference	95% CI		P value
PH (Ref: Normal)	$261,075	$252,675	$269,476				
Mild	$255,592	$247,528	$263,656	-$5,484	-$17,128	$6,161	0.356
Moderate	$256,422	$247,274	$265,571	-$4,653	-$17,073	$7,767	0.463
Severe	$258,138	$243,139	$273,136	-$2,938	-$20,128	$14,252	0.738

There was no significant difference in the mean cost between patients with normal, mildly elevated, moderately elevated, or severely elevated pulmonary pressure (p=0.810) (Table 6). Patients with severe PH had slightly lower costs than the other groups (mean: $235,556 vs 263,472: p-value=0.137).

**Table 6:** Multivariate Regression Comparing Mean Cost Among Patients With Normal, Mildly Elevated, Moderately Elevated, and Severely Elevated Pulmonary Pressure PH: Pulmonary hypertension

PH Groups	Coefficient	95% CI		P value	Predicted mean	95% CI		Difference	95% CI		P value
PH (Ref: Normal)					$263,472	$254,940	$272,004				
Mild	-0.03	-0.07	0.02	0.256	$256,782	$248,835	$264,730	-$6,690	-$18,231	$4,852	0.256
Moderate	-0.03	-0.08	0.02	0.205	$255,106	$245,950	$264,262	-$8,366	-$21,288	$4,555	0.204
Severe	-0.05	-0.12	0.02	0.142	$250,251	$235,556	$264,945	-$13,221	-$30,654	$4,212	0.137

## Discussion

Transcatheter aortic valve replacement has been the recommended therapy for moderate to severe symptomatic aortic stenosis in patients with moderate or high-surgical risk. Several studies were performed to outline the effects of different comorbidities on procedural outcomes. Pulmonary hypertension is commonly present in patients with aortic stenosis with prevalence recorded up to 75% in previous studies [5,7,8]. Pulmonary pressures were shown to improve post aortic stenosis intervention; however, it is noted that TAVR outcomes are impacted by the presence of pulmonary hypertension [6,9,10]. 

Previous studies demonstrated increased one-year mortality rates in patients with elevated pulmonary pressures post aortic valve replacements [11]. A study by Lindman et al. surprisingly showed increased hazard of death in females with PH, but no significant mortality changes were found in similar male patients [6]. In this study there was no association between hemodynamic factors and one-year mortality risk; other clinical factors however, such as oxygen-dependent lung disease, difficulty performing six-minute walk, or poor renal function independently increased that risk [6].

Our study categorized the severity of disease into normal pulmonary pressure, mildly, moderately, and severely elevated pressures for further risk stratification, then investigated the short-term outcomes for each group. Multivariable analysis was performed and did not demonstrate significant difference in overall cost of admission between all four groups. There was no association between the severity of pressure elevation and the odds of 30-day readmission rates, with P-values of 0.604, 0.673, and 0.202 in the mild, moderate, and severe PH groups respectively.

Univariate analysis showed that patients with severe PH spent significantly more hours in the ICU compared to patients with no or mild PH (mean: 18.2 hours, p-value<0.001). Patients with mild or moderate PH did not have a significant increase in their ICU stays.

Additionally, patients with severe PH had significantly higher odds of 30-day all-cause mortality (OR: 6.77, CI: 1.09-41.98: p-value=0.04) compared to patients with no or mild PH. All-cause mortality was previously established to be significant in the setting of persistent PH post aortic valve replacement. According to the 2018 study by Alushi et al., a higher risk of all-cause mortality was present in patients with residual PH post TAVR compared to those who had regression of PH postoperatively [5]. Similarly, the study published by Miyamoto et al. confirmed increased mortality risk in patients with new onset or persistent PH post intervention [12]. Here in this study, further risk stratification based on PH severity showed significantly worse outcomes only in the severe PH group. 

Although data showed significant reduction in PASP post TAVR in most patients with pulmonary hypertension and aortic stenosis, the findings of this study established the clinical impact of severely elevated pulmonary pressures on outcomes [5]. When evaluating patients for aortic valve replacements in the setting of significant PH, there may be benefits of optimizing their hemodynamics prior to undergoing the procedure, to minimize the risks of complications [13]. This, however, should not prevent or even delay the intervention for this group, given the substantial benefits including improving the functional status regardless of PH severity [11,14,15].

Limitations

The study has an inherent limitation of being retrospective, which limits the ability to create a causal relationship between the variable (pulmonary hypertension) and the outcomes. We had to exclude a lot of patients because of lack of complete data, which dropped the number of patients included in the study. We excluded the patients with systolic heart failure, however we cannot exclude patients with group 2 pulmonary hypertension secondary to diastolic heart failure, Although the incidence of diastolic heart failure leading to pulmonary hypertension is low, this can still lead to a potential sampling error.

## Conclusions

Severe pulmonary hypertension significantly increased the odds of 30-day mortality and ICU stay in TAVR patients. We did not see any significant difference in 30-day readmission and cost of admission based on PH severity.
